# High-Affinity Accumulation of a Maytansinoid in Cells via Weak Tubulin Interaction

**DOI:** 10.1371/journal.pone.0117523

**Published:** 2015-02-11

**Authors:** Victor S. Goldmacher, Charlene A. Audette, Yinghua Guan, Eriene-Heidi Sidhom, Jagesh V. Shah, Kathleen R. Whiteman, Yelena V. Kovtun

**Affiliations:** 1 Department of Cell Biology, ImmunoGen, Inc., Waltham, Massachusetts, United States of America; 2 Department of Systems Biology, Harvard Medical School, Boston, Massachusetts, United States of America; 3 Renal Division, Brigham and Women’s Hospital, Boston, Massachusetts, United States of America; University of Illinois at Chicago, UNITED STATES

## Abstract

The microtubule-targeting maytansinoids accumulate in cells and induce mitotic arrest at 250- to 1000-fold lower concentrations than those required for their association with tubulin or microtubules. To identify the mechanisms of this intracellular accumulation and exceptional cytotoxicity of maytansinoids we studied interaction of a highly cytotoxic maytansinoid, *S*-methyl DM1 and several other maytansinoids with cells. *S*-methyl DM1 accumulated inside the cells with a markedly higher apparent affinity than to tubulin or microtubules. The apparent affinities of maytansinoids correlated with their cytotoxicities. The number of intracellular binding sites for *S*-methyl DM1 in MCF7 cells was comparable to the number of tubulin molecules per cell (~ 4–6 × 10^7^ copies). Efflux of ^3^ [H]-*S*-methyl DM1 from cells was enhanced in the presence of an excess of non-labeled *S*-methyl DM1, indicating that re-binding of ^3^ [H]-*S*-methyl DM1 to intracellular binding sites contributed to its intracellular retention. Liposomes loaded with non-polymerized tubulin recapitulated the apparent high-affinity association of *S*-methyl DM1 to cells. We propose a model for the intracellular accumulation of maytansinoids in which molecules of the compounds diffuse into a cell and associate with tubulin. Affinities of maytansinoids for individual tubulin molecules are weak, but the high intracellular concentration of tubulin favors, after dissociation of a compound-tubulin complex, their re-binding to a tubulin molecule, or to a tip of a microtubule in the same cell, over their efflux. As a result, a significant fraction of microtubule tips is occupied with a maytansinoid when added to cells at sub-nanomolar concentrations, inducing mitotic arrest and cell death.

## Introduction

A number of tubulin-binding agents are in clinical use either as anticancer drugs or as tumor-targeted antibody-drug conjugates [1–3]. Maytansine and its derivatives, maytansinoids, accumulate in cells and induce mitotic arrest and subsequent cell death [4,5]. These compounds have affinity to both nonpolymerized tubulin (αβ-heterodimer), and to the tips of microtubules, and are believed to arrest mitosis through their interaction with the mitotic spindle microtubule tips, which leads to suppression of the dynamic instability of the microtubules [4,6]. Among the hypothetical explanations for the high cytotoxicity of tubulin-binding agents are: (i) their ability to accumulate inside cells at low concentrations, seemingly lower than their affinities to both tubulin and microtubules, and (ii) their ability to induce cell death by binding to only a fraction of microtubules, because cells may be arrested at mitosis when only one or several chromosomes fail to segregate due to suppression of dynamic instability of their mitotic spindle microtubules [4,5,7]. While interactions of maytansinoids with tubulin and microtubules in solution have been examined [6,8], the reasons for their ability to accumulate in cells at concentrations lower than those at which they associate with either tubulin or microtubules have not yet been elucidated. To better understand the reasons for this exceptional ability of these tubulin binders to accumulate in cells, and, in particular, what makes them so cytotoxic and effective as cytotoxic moieties in antibody-drug conjugates [9], we studied the interaction of the functionally active maytansinoid *S*-methyl DM1 (*N* ^2′^-deacetyl-*N* ^2′^-[3-(methylthio)-1-oxopropyl]-maytansine) and several other maytansinoids with cultured cells and with tubulin-loaded liposomes.

## Materials and Methods

### Materials

*S*-methyl DM1 and other maytansinoids were synthesized as described previously [6]. ^3^[H]-*S*-methyl DM1 was synthesized at American Radiolabeled Chemicals. 1,2-dioleoyl-sn-glycero-3-phosphocholine in chloroform (Avanti Polar Lipids) was stored under argon at-20^o^ C. ^3^[H]-paclitaxel was from Moravek Biochemicals. MCF7 cell tubulin and bovine brain tubulin (>99%; isolated by the method described in [10]) were purchased from Cytoskeleton, Inc. Paclitaxel was from Tocris; nocodazole, vinblastine sulfate, demecolcine, monoclonal anti-α-tubulin clone B-5–1–2 murine IgG1 T6074, RIPA buffer from Sigma; Halt Protease & Phospatase Inhibitor Cocktail (no EDTA or other chelators) was from Thermo Scientific; DilC18(3) and MagicMark XP protein size marker were purchased from Invitrogen; peroxidase-conjugated AffiniPure F(ab’)2 fragment goat anti mouse IgG (H+L) 115–036–062 was purchased from Jackson Labs; ECL Advanced Blocking Agent CPK1075 was purchased from GE Healthcare.

### Cell lines

MCF7 human epithelial breast adenocarcinoma (HTB-22), Farage human non-Hodgkin’s B cell lymphoma (CRL-2630), and COLO 205 human epithelial colorectal adenocarcinoma (CCL-222) cell lines were obtained from the American Type Culture Collection (ATCC); ECFP-PTK-2 cells, a clonal subline of PtK2 potoroo kidney epithelial cell line (ATCC CCL-56) stably expressing enhanced cyan mutant of green fluorescent protein (ECFP)-tubulin fusion protein were described in [11]; HL60/QC and BJAB-E10 human cell lines are sublines generated at ImmunoGen from HL-60 human acute promyelocytic leukemia (ATCC, CCL-240), and from BJAB human Burkitt lymphoma cell line (a gift from E. Kieff, Harvard Medical School; Leibniz Institute DSMZ-German Collection of Microorganisms and Cell Cultures no.: ACC 757); IGROV-1 human ovarian cancer cell line was purchased from the Division of Cancer Treatment and Diagnosis, National Cancer Institute, Frederick, MD, HSC-2 human mouth squamous carcinoma cell line from the Japan Health Sciences Foundation, and HPB-ALL human T cell leukemia cell line from Leibniz Institute DSMZ-German Collection of Microorganisms and Cell Cultures. All cell lines were cultured in the media recommended by the providers in a humidified incubator at 37^o^ C, 6% CO_2_.

### Fluorescence correlation spectroscopy (FCS)

ECFP-PtK2 cells were cultured in glass-bottom 35mm dishes with No. 1.5 cover glass (MatTek Corporation) in Eagle’s Minimum Essential medium supplemented with 10% fetal bovine serum (FBS), penicillin, and streptomycin. This medium was replaced by CO_2_-independent medium (Invitrogen) supplemented with 5% FBS for live cell imaging and FCS measurements. Fluorescent images were taken on a Nikon Eclipse Ti inverted microscope system (Nikon) heated with an enclosed chamber (In Vivo Scientific). For each experiment, a ten-frame movie was collected with a PlanApo 60XA (NA = 1.4, Ph3 DM) oil objective through CFP HQ filter cube (Chroma). Images were captured to a cooled charge-coupled device camera (CoolSNAP HQ, Roper Scientific) with exposure time 200 ms at 2-by-2 binning. The microscopy system was controlled by Elements software (Nikon). The ten frames in each movie were averaged out to decrease the background noise through the ImageJ software (http://rsbweb.nih.gov/ij). We measured fluctuations in tubulin fluorescence within the cell to monitor tubulin oligomers generated by depolymerization [11]. Cells were exposed to the compounds at the following concentrations/exposure times: nocodazole at 0.3 μM for 60 min, *S*-methyl DM1 at 0.2 μM for 90 min, demecolcine at 1.6 μM for 90 min, and vinblastine at 1 μM for 90 min. Multiphoton FCS was carried out on a custom built setup based on a Nikon TE2000 microscope. For ECFP excitation, a collimated 850nm IR laser beam (Mai Tai, Ti:Sapphire laser with 80MHz and ~100 fs pulse width, Spectra-Physics, CA, USA) was aligned into Nikon 100X Plan Apochromat oil immersion objective (N.A. = 1.4) with back aperture slightly overfilled, creating a diffraction-limited focal spot. The laser power was controlled below 2 mW (measured outside the microscope) to avoid photobleaching of the fusion protein and cellular photodamage. The collected emission fluorescence was passed through band-pass filters (HQ485/70m-2p for ECFP (Chroma) to a photomultiplier tube (H7421, Hamamatsu, Japan). The cells were maintained in an aluminum chamber with temperature-controlled water circulation system. Each autocorrelation curve measured in cytoplasm was collected for 30 seconds using Flex02–01D/C correlator (correlator.com) and transferred to a personal computer through a high speed USB port. To construct an average curve, five FCS curves were run at each position in a single cell. FCS curves were analyzed by custom-written Matlab code (Mathworks Inc, Waltham, MA) using a nonlinear least-squares fitting algorithm. The fitting formula for single-component diffusion is adapted from:

$$G\left(t\right)=\frac{1}{N}{\left(1+\frac{t}{\tau_{D}}\right)}^{-1}{\left(1+\frac{t}{\omega^{2}\tau_{D}}\right)}^{-1/2}$$

where <*N*> is the average particle number of species in the sampling volume; *t* is the correlation or delay time (x-axis of the autocorrelation function); *τ*_*D*_ is the residence time of species within the sampling volume, with *τ*_*D*_ = *ω* ^*2*^_*xy*_ */8D*, where *D* is the diffusion coefficient of the species, and *ω* = *ω*_*z*_ */ω*_*xy*_ is the aspect ratio of the sampling volume. Before the fit analysis, raw FCS autocorrelation curves were denoised by a moving average filter of window span 5. For single curve fit, each FCS was fitted by the formula above and the resulting diffusion property, molecular number and brightness were compared with each condition. For the averaged curve fit, the five FCS curves at each measurement position were averaged before fitting. Curves shown in the S1 Fig. have been normalized to permit comparison of diffusion times, but <*N*> and τ_D_ values were fit to the raw autocorrelation data. Brightness analysis was conducted by dividing total fluorescent counts by the average particle number <*N*> to yield fluorescent counts per diffusing particle.

### Cytotoxicity assays

MCF7 cells were plated onto 6-well tissue culture plates (1000 cells/well) in 2 mL culture medium and incubated for 24 h at 37^o^ C. The medium was then replaced with 2 mL of medium containing a specified concentration of a cytotoxic compound, or medium alone (to determine the plating efficiency of the cells). Following 4-h incubation, the plates were rinsed with culture medium and cultured in compound-free medium for 6 days to allow colony formation. Colonies (≥ 50 μm average diameter corresponding to ~ 50 cells or more) were visualized by staining formaldehyde-fixed monolayers with Crystal violet, and counted using a GelCount Colony Counter (Oxford Optronix Ltd.).

### Computational model for enrichment of a tubulin-binding compound within a compound-permeable sphere containing tubulin

This computational model assumes a spherical geometry of a cell (aqueous phase surrounded by a membrane) in which there is complex formation of intracellular tubulin with a membrane-permeable drug. The system consists of three species: the intracellular tubulin ***t***, a membrane permeable compound, ***c*** and the compound-tubulin complex ***c*:*t*** (Table 1). The compound is assumed to be at sufficient amount outside the cell (at the concentration of [*c*]_*out*_ such that the amount that diffuses into the cell (to the intracellular concentration [*c*]_*in*_) is insignificant in comparison with the total amount outside the cell, thus maintaining a constant outside concentration. The model was solved as a set of partial differential equations (Table 2) evaluated using MATLAB’s *pdepe*-function, assuming spherical symmetry. [*c*]_*out*_ was set to 10 nM, and the radius of the cell was set to 5 μm. At the cell boundary, the tubulin and the compound-tubulin complex species are subject to a no flux condition preventing tubulin exit. The diffusion of the compound into the cell is controlled by a permeability coefficient, *D*_*m*_. This permeability coefficient did not affect equilibrium concentrations, only the time to reach equilibrium, and was set to 0.5. The diffusion constant of the compound was set to 200 μm^2^/s and the diffusion constants of tubulin and compound-tubulin complex were set to 40 μm^2^/s, based on previous work [11]. The simulation was run to steady state and then the species concentrations were recorded to calculate the [*c*]_*in*_/[*c*]_*out*_) enrichment ratio (or partition coefficient). The intracellular concentration of tubulin was varied from 1 nM to 1 mM. The equilibrium dissociation constant K_d_ of the compound-tubulin complex was also varied from 1 nM to 1 mM. The interaction between the compound and tubulin is controlled by the k_on_ and k_off_ rate constants. The model was run using two k_on_ values: a) a diffusion-limited association was assumed by setting k_on_ to 1 nM^-1^s^-1^, and then k_off_ was calculated based on the desired K_d_, or b) the k_on_ was set to 18 nM^-1^s^-1^, and then k_off_ was calculated based on the desired K_d_. Simulations using these two k_on_ values affected the time to reach steady state, but produced the same steady state partitioning coefficient, as would be expected for an equilibrium phenomenon.

**Table 1:** Compound-tubulin reaction.

Chemical reaction	Parameters
$c+t\overset{k_{on}}{\underset{k_{off}}{⇌}}c:t$	*k*_*on*_: either 1 nM^-1^s^-1^, or 18 nM^-1^s^-1^; *k*_*off*_; varies

**Table 2:** Reaction-diffusion equations.

	Equation
Membrane diffusion and cell boundary conditions	$D_{d}\frac{\partial \left[c\right]}{\partial x}=D_{m}\left({\left[c\right]}_{out}-{\left[c\right]}_{in}\right),\tilde{D}\frac{\partial \left[\tilde{u}\right]}{\partial x}=0$,
Reaction kinetics	$\frac{\partial \left[\tilde{u}\right]}{\partial t}=\tilde{D}\nabla^{2}\left[\tilde{u}\right]+S\left(\left[\tilde{u}\right]\right)$

Description: [*c*]_*in*_: compound concentration inside the cell, [*c*]_*out*_: compound concentration outside the cell, *D*_*d*_: diffusion constant of the compound, *D*_*m*_: permeability of the membrane to compound, $\tilde{D}$: is the vector of diffusion constants.

### Cellular tubulin quantitation

3 x 10^6^ cells were washed in PBS, resuspended in 0.2 mL RIPA buffer freshly supplemented with protease inhibitors cocktail, and mixed with 22 μl 10x DNAse I buffer (New England Biolabs), and 10 μl DNAse I (Roche). Following 10 min incubation at room temperature, 290 μL NUPAGE LDS sample buffer (Invitrogen) and 56 μL β-mercaptoethanol were added; the mixture was incubated at 85°C and run on a NUPAGE 4–12%/MOPS gel (Invitrogen). Tubulin isolated from MCF7 cells (Cytoskeleton) was used as a reference standard. Western blot was done with anti-α-tubulin (1:1000), developed with peroxidase-conjugated AffiniPure F(ab’)2 fragment goat anti mouse IgG and visualized by enhanced chemiluminescence.

### Preparation of liposomes loaded with tubulin

All buffers were degassed prior to the experiments. Immediately before an experiment, bovine brain tubulin was dissolved in PBS/25 mM EGTA (to prevent assembly into microtubules), centrifuged for 10 min at 16000 x g at 4° C to remove possible aggregates, desalted on a micro Bio-Spin column with Bio-Gel P-6 (Bio-Rad) pre-equilibrated with PBS, and its concentration assessed by OD_280_ (molar extinction coefficient 1.015 x 10^5^). 3.75 mg (0.15 mL) of 1,2-dioleoyl-sn-glycero-3-phosphocholine in chloroform was mixed with the fluorescent lipid marker DilC18(3) in methylene chloride at 100:1 molar ratio, dried under argon in a glass vessel, mixed with 0.25 mL tubulin solution in PBS, vortexed, the suspension was left hydrating for 30 min at room temperature, and vortexed again. Liposomes were then formed using mini-extruder (Avanti Polar Lipids) and Whatman Nucleopore Track-Etch Membrane 800282 (19 mm, 0.4 μm) in accordance with the manufacturer’s recommendations. The liposome suspension was mixed with 1.4 mL 0.15 M NaCl and centrifuged at 16000 x g for 10 min at 4° C. The pellet (liposomes) was resuspended in 2 mL 0.15 M NaCl, centrifuged again as above, then resuspended in 0.2 mL 0.15 M NaCl and run at room temperature through a NAP-5 column (GE Healthcare) that had been equilibrated with 0.15 M NaCl. The liposome suspension (0.5 mL) was mixed with 1.5 mL 0.15 M NaCl, centrifuged again as above, resuspended in 0.2 mL PBS, and the concentration of tubulin in the suspension was determined as follows. Serial 1:1 dilutions of liposome suspensions and of bovine brain tubulin of a known concentration were run on a Novex/Invitrogen NuPAGE SDS-PAGE Gel, stained by SimplyBlue Coomassie (Invitrogen) and compared. At least two independent gels were run and then average tubulin concentrations were calculated. The relative concentrations of lipids in liposomal suspensions prepared for different experiments were compared by the fluorescence of the DilC18 marker (excitation 530 nm/emission 570 nm) using a fluorescence plate reader and black flat-bottom 96-well plates. In initial experiments we confirmed that the fluorescent signal was directly proportional to the amount of liposomes per well.

### Binding of ^3^[H]-labeled compounds to liposomes

0.2 mL liposomal suspension was mixed with 0.2 mL PBS supplemented with 1 mg/mL bovine serum albumin (PBS/BSA), and 12 μL of the suspension was mixed with various concentrations of a tritiated compound, and, in some samples, an excess of the non-labeled compound, and with PBS/BSA to the final volume of 40 μL. Tritiated and non-labeled *S*-methyl DM1 and paclitaxel stock solutions were in ethanol. Prior to mixing these stock solutions with an aqueous buffer they were mixed with an equal volume of dimethyl sulfoxide to prevent precipitation. The concentrations of the organic solvents in the samples never exceeded 1% volume/volume, and appropriate amounts of these solvents were added to control samples. The concentration of excess non-labeled *S*-methyl DM1 and paclitaxel used in these experiments were 6 μM and 3.4 μM, respectively. After 1.5-h incubation at 37°C liposomes were separated from the non-bound compound by running the samples at room temperature through NAP-5 columns that had been equilibrated with PBS/BSA. The beta-radioactivity of the samples was then determined as described above. In initial experiments, we found that ^3^[H]-*S*-methyl DM1 that was associated with tubulin-loaded liposomes after running the samples through a NAP5 column were fully retained after running the liposomes through a second NAP5 column, indicating that under the experimental conditions the liposome-bound compounds were fully retained during the separation of bound from non-bound material.

### Determination of the volume of cells

A cell suspension containing 3 x 10^7^ to 3 x 10^8^ cells (as determined on a Coulter Counter) was centrifuged at 256 x g, the liquid was aspirated, and the total cell volume was determined by weight (assuming the density of 1 g/mL).

### Binding of ^3^[H]-S-methyl DM1 and ^3^[H]-paclitaxel to cells

Cell suspensions (2 x 10^5^ cells/mL) were incubated at 37° C in culture medium (8 mL) containing a ^3^[H]-labeled compound. Some samples also contained an excess of the respective non-labeled compound (5 μM *S*-methyl DM1, or 50 μM paclitaxel). At various times, 1-ml samples of cell suspension were centrifuged, the cell pellet was suspended with 10 ml of culture medium, centrifuged again, the pellet was lysed with 0.4 ml 50 mM Tris/0.2 M NaCl/15 mM EDTA, pH 8.0, containing 1% (v/v) Triton X-100, mixed with Ultima Gold scintillation fluid (Perkin-Elmer Life Sciences), and the beta-radioactivity was measured using a Packard Tri-Carb 2900 TR liquid scintillation analyzer. In initial experiments, we determined the length of exposure sufficient to reach the binding equilibrium for each cell line tested, and each concentration of the tritiated compound. From the data, and the specific activity of the tritiated compound, we calculated the number of molecules bound per cell, and the number of molecules remaining in the supernatants. GraphPad Prism Software was used to determine the K_d_ values and the number of binding sites. To measure efflux of ^3^[H]-*S*-methyl DM1 from MCF7 cells, adherent cells (1.3 x 10^5^ cells/cm^2^) were incubated for 2 h at 37°C in culture medium (4 mL) containing 3.3 nM ^3^[H]-*S*-methyl DM1. The medium was replaced with fresh culture medium (4 mL), containing either no drug, or, in some samples, non-labeled *S*-methyl DM1 (0.33 μM). The cells were incubated at 37°C, then harvested by trypsinization over the course of incubation, and processed as above for measurement of radioactivity.

### Competition binding of ^3^[H]-S-methyl DM1 with maytansinoids

Cells (2 x 10^5^ cells/sample) were incubated for 3 h at 37° C in culture medium (2 mL) containing a mixture of 0.1 nM ^3^[H]-*S*-methyl DM1 and one of the non-labeled competitors, S-methyl DM1, maytansine, dithio-methyl DM1, or dithio-methyl-2’R DM1. Following the incubation, cells were centrifuged, and the supernatant was removed. The cell pellets were resuspended with culture medium (10 mL), centrifuged again, and the cell pellet was lysed and processed as above. The EC_50_ values (50% inhibition, average of two independent experiments) were then determined using GraphPad software (non-linear regression curve fit).

## Results

### S-methyl DM1 accumulates in cells with high apparent affinity

**Fig 1:**
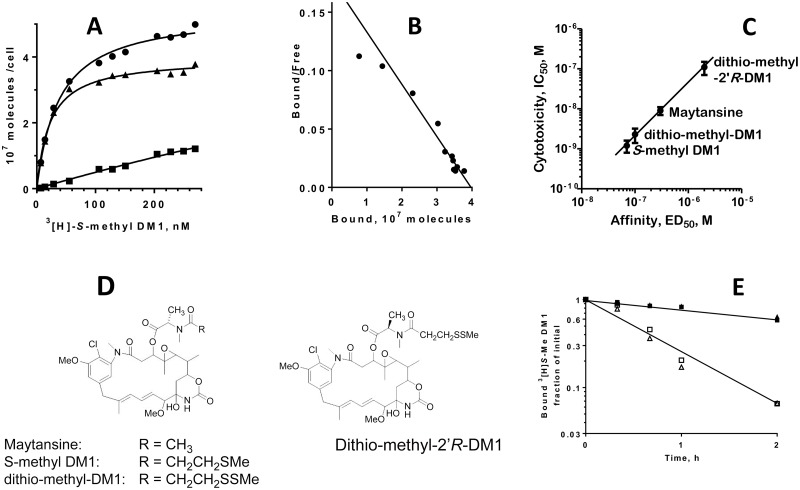
Interaction of maytansinoids with MCF7 cells. A. Binding of the maytansinoid ^3^[H]-*S*-methyl DM1 to the cells, a representative experiment. Cells were incubated in culture medium with various concentrations of ^3^[H]-*S*-methyl DM1 at 37° C for 4 h (equilibrium was reached within 4 h) with (squares) or without (circles) an excess (5 μM) of non-labeled *S*-methyl DM1, washed two times with medium, and cell-associated radioactivity was measured on a scintillation counter. The specific binding (triangles) was calculated as the difference between the two values. Cell-associated ^3^[H]-*S*-methyl DM1 was fully retained by cells after multiple washes, and thus the equilibrium was not distorted during the washes. B. Scatchard plot of A. C. The relationship between the cytotoxicity (IC_50_) of a maytansinoid and its apparent affinity (ED_50_) to cells. The IC_50_ values were determined in clonogenic assays after a 4-h exposure of cells to a maytansinoid. Each point (the mean ± standard error) is the result of three independent experiments. The ED_50_ is a concentration of a maytansinoid that decreased the binding of a trace amount of ^3^[H]-*S*-methyl DM1 by 50% in a competition binding assay. D. Structures of the maytansinoids. E. Efflux of ^3^[H]-*S*-methyl DM1 from MCF7 cells at 37° C in medium (filled symbols), or in the presence of an excess of non-labeled *S*-methyl DM1 (open symbols). Triangles and squares represent two independent experiments.

**Fig 2:**
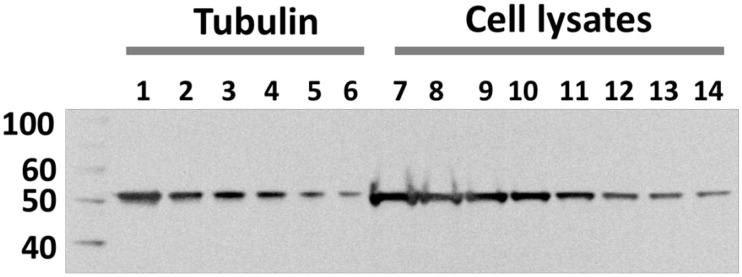
Tubulin contents in MCF7 cells. Tubulin isolated from MCF7 cells used as a standard (lanes 1–6; 278, 185, 130, 93, 56, 37 ng/lane, respectively), or MCF7 cell lysates (lanes 7–14; 7.7, 5.2, 3.9, 2.6, 1.8, 1.0, 0.77, 0.52 x10^4^ cells/lane, respectively) were resolved by SDS-PAGE. Three independent experiments produced similar results.

To explore the reasons for the accumulation of maytansinoids in cells, we examined the apparent affinity of a highly cytotoxic maytansinoid, *S*-methyl DM1 to cells. Equilibrium binding curves of ^3^[H]-*S*-methyl DM1 to MCF7 cells at 37° C (Fig. 1A, 1B) revealed a single class of high-affinity binding sites, (3.2 ± 0.4) x 10^7^ per cell with an apparent equilibrium dissociation constant K_d_ of 29 ± 6 nM (n = 3). *S*-methyl DM1 had comparable affinities to other tested cell lines with apparent K_d_ values between 11 to 31 nM (Table 3). The affinity of *S*-methyl DM1 to cells was markedly higher than its affinity to nonpolymerized tubulin (αβ-heterodimer), or to the tips of reconstructed microtubules in cell-free systems (apparent K_d_ of 0.4 to 0.9 μM, and 0.1 μM, respectively [6,8]). The apparent affinities of *S*-methyl DM1 and several other maytansinoids and maytansine to MCF7 cells correlated with their cytotoxicities (Fig. 1C; the structures are shown in Fig. 1D), indicating that the affinity of a maytansinoid to cells is a determinant of its cytotoxic potency. In particular, dithio-methyl-2’*R* DM1, a stereoisomer that bound to cells only weakly (30-fold weaker than *S*-methyl DM1) was 88-fold less cytotoxic. Efflux of ^3^[H]-*S*-methyl DM1 from MCF7 cells was slow: 65 ± 3% (n = 2) of the compound retained in cells after a 2-h incubation in medium at 37°C. The rate of efflux increased 3-fold in the presence of an excess of non-labeled *S*-methyl DM1 (Fig. 1E), indicating that re-binding of ^3^[H]-*S*-methyl DM1 to its intracellular binding sites contributed to its intracellular retention. We estimated the total number of tubulin molecules per MCF7 cell as 4 to 6 x 10^7^ (Fig. 2), which was consistent with the previously published data [12,13], and similar to the number of intracellular maytansine binding sites. The number of microtubule tips to which maytansinoids also bind [6] would be orders of magnitude lower [12]. Microtubules, therefore, are unlikely to constitute a significant fraction of intracellular binding sites for *S*-methyl DM1. A large fraction of tubulin in cells is apparently present in the state of non-assembled monomeric or dimeric heterodimer [11,13–16], by various estimates 20–70%, possibly depending on the cell type.

**Table 3:** Binding of ^3^[H]-*S*-methyl DM1 to human cell lines.

Cell line	10^7^ Binding sites/cell	K_d_, nM
MCF7	3.2 ± 0.4	29 ± 6
BJAB	2.8 ± 0.1	20 ± 5
COLO 205	2.0 ± 0.2	21 ± 4
Farage	0.7 ± 0.3	10.7 ± 0.8
HL-60	1.1 ± 0.3	23 ± 8
HPB-ALL	0.72 ± 0.01	28.6 ± 0.3
HSC-2	4.6 ± 0.3	31 ± 2
IGROV-1	2.7 ± 0.3	29 ± 4

The values are presented as means ± standard error. Each value is a result of two independent experiments, except for MCF7 cells for which the values were determined in three independent experiments.

### S-methyl DM1 does not induce oligomerization of tubulin in cells

**Fig 3:**
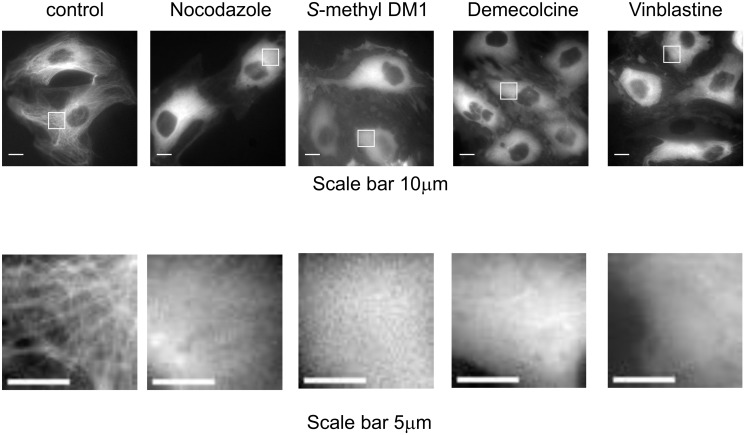
Fluorescent microscopy photographs of ECFP-PTK2 cells treated with various microtubule depolymerizing agents. Each compound was added into culture medium with the following final concentration and incubated for the following time period: nocodazole (0.3 μM for 60 min), *S*-methyl DM1 (0.2 μM for 90 min), demecolcine (1.6 μM for 90 min) and vinblastine (1 μM for 90 min). Scale bars are 10 μm for main images and 5 μm for sub-region images.

**Fig 4:**
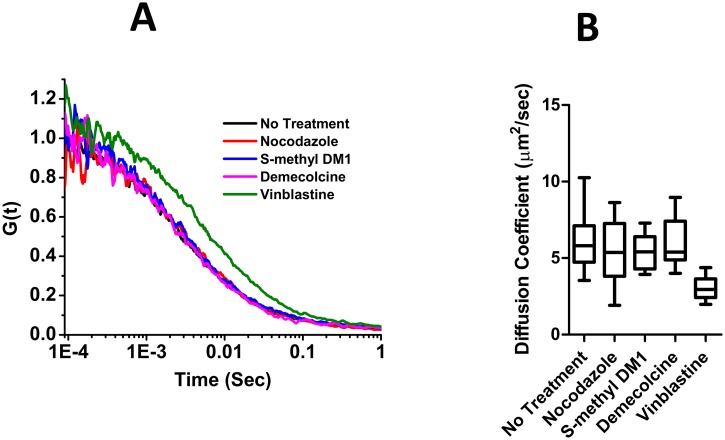
*S*-methyl DM1 does not induce oligomerization of ECFP tubulin in ECFP-PTK2 cells. A. Intracellular fluorescence autocorrelation curves for ECFP tubulin in ECFP-PTK2 cells treated with various microtubule depolymerizing agents. Each curve was the average of individual cell measurements: a total of 30 cells and 144 measurements were collected for average for non-treated cells, a total of 12 cells and 60 measurements for nocodazole, a total of 9 cells and 54 measurements for both *S*-methyl DM1 and demecolcine, and a total of 11 cells and 52 measurements for vinblastine. B. Diffusion coefficients of cytoplasmic tubulin obtained from the FCS measurements in living cells treated with microtubule depolymerizing agents. Whiskers indicate minimum to maximum and box median value (line within the box).

To determine if incubation of cells with *S*-methyl DM1 leads to formation of tubulin oligomers which might potentially serve as binding sites, we examined the diffusion of non-assembled tubulin in cells exposed to *S*-methyl DM1 by fluorescence correlation spectroscopy (FCS). In these experiments we used ECFP-PtK2 cells which express tubulin fused to cyan fluorescent protein It has been established using FCS that cytoplasmic unincorporated tubulin in PtK2 cells has a diffusion coefficient (~6.0 μm^2^/s) consistent with a monomeric or dimeric αβ-heterodimer, but not with higher oligomeric species [11]. Formation of larger tubulin oligomers would result in a smaller diffusion coefficient. We exposed cells to *S*-methyl DM1, or, as a positive control, to another microtubule-depolymerizing agent, vinblastine [17], which induces formation of tubulin oligomers in cell-free systems [18–20]. For comparison, we also exposed cells to other microtubule-depolymerizing agents, nocodazole or demecolcine, at 37° C. For each of these agents we chose a concentration (see Methods), that caused dissasembly of microtubules (Fig. 3), and could potentially lead to formation of tubulin oligomers. The fluorescence time autocorrelation curves, which reflect the average number of molecules contained within the focal volume (Table 4), and the diffusion coefficients of the major fraction of fluorescent tubulin in cells that were exposed to *S*-methyl DM1, nocodazole or demecolcine (Fig. 4) were similar to those in non-treated cells, indicating that fluorescent tubulin did not form any oligomers. This result was consistent with previous reports that only minimal cell-free tubulin aggregation occured in the presence of *S*-methyl DM1 and maytansine [6,8]. The molecular number of molecules that rapidly diffused increased under treatment with nocodazole, demecolcine, or *S*-methyl DM1, when compared to untreated cells (Table 4) supporting the observation of microtubule depolymerization. The behavior of fluorescent tubulin in vinblastine-treated cells suggested that in these cells tubulin was in an oligomerized state, in accord with the previous observations in cell-free systems [18–20]. The FCS brightness analysis did not reveal an increase in tubulin stoichiometry under vinblastine treatment indicating that the tubulin-vinblastine complexes may interact with non-tubulin moieties within the cell. Taken together, our data suggested that in cells, non-assembled tubulin constitutes the bulk of the binding sites for *S*-methyl DM1, but we still needed to find out why the apparent affinity of *S*-methyl DM1 to cells was markedly higher than its respective affinity to nonpolymerized tubulin, or to the tips of microtubules.

**Table 4:** FCS Autocorrelation analysis of ECFP-tubulin in PTK2 cells treated with microtubule depolymerizing agents.

	No treatment	Nocodazole	*S*-methyl DM1	Demecolcine	Vinblastine
Molecular number	8 ± 1	17 ± 3	18 ± 3	11 ± 3	6 ± 1
Dwell time, ms	3.0 ± 0.2	3.6 ± 0.5	3.2 ± 0.2	3.0 ± 0.3	5.8 ± 0.4
Number of cells	29	12	9	9	11

Each compound was added into culture medium at the following final concentration: nocodazole (0.3 μM), *S*-methyl DM1 (0.2 μM), demecolcine (1.6 μM) and vinblastine (1 μM). Average molecular number (ECFP-tubulin) and average residence time (inversely related to diffusion constant) were determined by fitting time autocorrelation curves (Fig. 4A) to a 3D diffusion model (see Methods).

### S-methyl DM1 binds to tubulin-loaded liposomes with an affinity comparable to its affinity to cells

**Fig 5:**
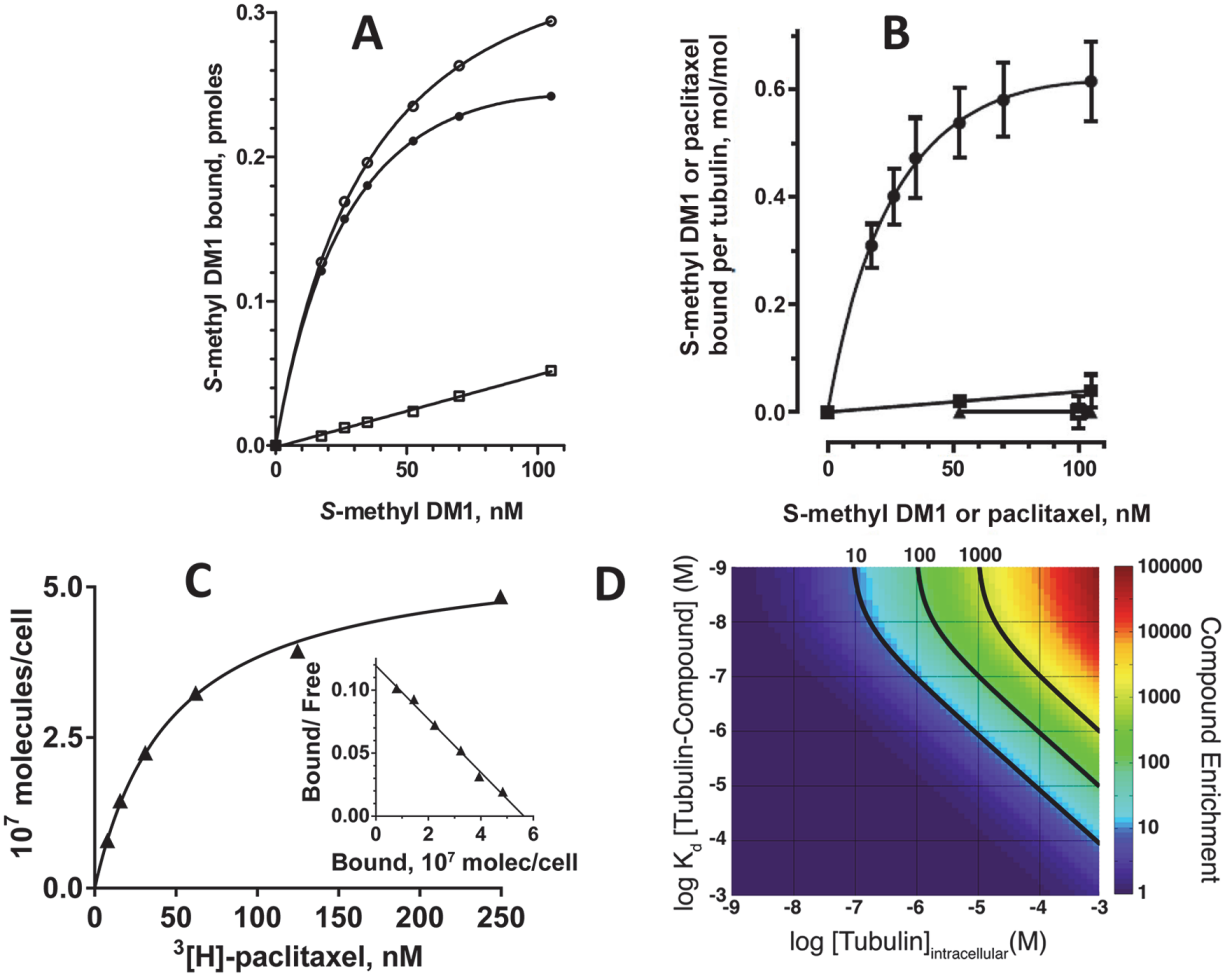
Accumulation of *S*-methyl DM1 in model systems. A. Binding of ^3^[H]-*S*-methyl DM1 to tubulin-containing liposomes. In the representative experiment, liposomes formed in the presence of 97 μM tubulin were incubated with various concentrations of ^3^[H]-*S*-methyl DM1 at 37° C for 1.5 h (equilibrium was reached within 1 h) with (open squares) or without (open circles) an excess (6 μM) of non-labeled *S*-methyl DM1, then separated from non-bound ^3^[H]-*S*-methyl DM1 on a Sephadex G25 desalting column, and liposome-associated radioactivity was measured on a scintillation counter. The specific binding (filled circles) was calculated as the difference between the two values. B. Binding of ^3^[H]-*S*-methyl DM1 to tubulin-containing liposomes formed in the presence of a high (97 μM; circles) or a low (7 μM, squares) concentration of tubulin, or to tubulin in solution (triangles). The experimental conditions are similar to those in A. Also shown is specific binding of ^3^[H]-paclitaxel to to liposomes formed in the presence of 97 μM tubulin (open square). Each point (the mean ± standard error) is the result of two independent experiments. C. Specific binding of ^3^[H]-paclitaxel to MCF7 cells and the Scatchard plot, a representative experiment. D. Computation of accumulation of a tubulin-binding compound inside a cell containing tubulin (see text).

The spatial distribution of tubulin in cell culture is heterogeneous; tubulin is localized in cells, but not in the surrounding medium, while when the same amount of tubulin is dissolved in an aqueous solution, it is evenly distributed at a lower concentration. We used liposomes containing purified bovine brain tubulin to mimic this heterogeneity of spatial distribution of tubulin in cell cultures, and to examine the affinity of *S*-methyl DM1 to tubulin in the absence of other cellular proteins that might be involved in the formation of intracellular binding sites. We entrapped 97 μM tubulin dissolved in a buffer devoid of GDP/GTP and Ca^2+^, which inhibited its polymerization [20–22] into dioleolyl phospatidylcholine liposomes, separated these liposomes from the non-entrapped tubulin, and examined equilibrium binding of ^3^[H]-*S*-methyl DM1 to these liposomes at 37° C (Fig. 5A). ^3^[H]-*S*-methyl DM1 accumulated in tubulin-containing liposomes with a high apparent affinity (K_d_ of 21.5 ± 0.07 nM, n = 2). In contrast, binding of ^3^[H]-*S*-methyl DM1 to either a similar amount of tubulin in liposomes made in the presence of a 14-fold lower concentration of tubulin (7 μM), or to a similar amount of tubulin in solution (Fig. 5B), or to a similar amount (by lipid, Methods) of empty liposomes was negligible, in accord with the previously published data that *S*-methyl DM1 bound to tubulin only at concentrations of *S*-methyl DM1 which were markedly higher than those used in our experiments [6,8]. To verify that tubulin in liposomes was non-polymerized, we examined the affinity of ^3^[H]-paclitaxel to liposomes loaded with 97 μM tubulin, as well as to MCF7 cells. Paclitaxel does not bind to non-polymerized tubulin, but it triggers tubulin polymerization and binds to microtubule-assembled tubulin at ~ 1:1 molar ratio [23,24]. The buffer that we used in liposomes inhibits polymerization of tubulin [20–22], while a large fraction of cellular tubulin in the presence of paclitaxel is likely to be assembled into microtubules [25]. We expected that paclitaxel would not bind to liposomes, but would accumulate in cells by binding to assembled tubulin, as previously reported [26]. As shown in Fig. 5C, ^3^[H]-paclitaxel bound to MCF7 cells with a K_d_ of 60 ± 10 nM. The number of paclitaxel binding sites/cell was (5.72 ± 0.08) x 10^7^, i.e. similar to the total cellular tubulin content. In contrast to ^3^[H]-*S*-methyl DM1, ^3^[H]-paclitaxel did not specifically bind to tubulin-containing liposomes (Fig. 5B), consistent with its inability to associate with non-polymerized tubulin [23,24]. Based on cell volume, the intracellular concentration of tubulin in cells tested in this study is between 12 to 18 μM (Table 5), similar to previous estimates for tubulin in CA46 cells (48 μM) [12]. Concentration of tubulin in the aqueous intracellular phase is likely to be significantly higher than its overall concentration in cells, because tubulin is expected to be excluded from much of the intracellular space occupied by other intracellular proteins and organelles. The higher concentration of tubulin in the aqueous intracellular space might be one reason why cells accumulated *S*-methyl DM1, while liposomes made in the presence of 7 μM tubulin did not. In addition, cells are much larger than liposomes. At the same internal concentration of tubulin, e.g. 7 μM, a 5-pL cell would contain ~ 2 × 10^7^ molecules of tubulin, while a 0.03 fL (0.4 μm diameter) liposome would contain only ~ 130 molecules. The latter number may be too low to favor rebinding of a tubulin-binding compound over its efflux.

**Table 5:** Concentration of tubulin in cells.

Cell line	Cell volume, pL	Tubulin, μM
MCF7	6.8 ± 0.5	12
BJAB	2.0 ± 0.3	18
HSC-2	8.2 ± 0.4	17
IGROV-1	5.3 ± 0.5	12

The intracellular concentration of tubulin was estimated using its intracellular content, and the cell volume (Methods). Each point (mean ± standard error) is the result of two independent experiments.

### A computation model predicts accumulation of low-affinity tubulin binding compounds in cells

We used a set of partial differential equations (Table 2) to compute how much enrichment of a tubulin-binding compound one could expect to generate. The model included a spherical aqueous phase surrounded by a membrane, loaded with tubulin, and external aqueous space with a membrane-permeable compound, which has affinity for tubulin. The model for an extracellular compound concentration of 10 nM was run until steady state, and the enrichment ratio for the compound (the intracellular concentration/extracellular concentration ratio) was calculated over a wide range of compound-tubulin affinities and intra-cellular tubulin concentrations. The resulting heat-diagram shown in Fig. 5D predicts ~ 20 to 200-fold intracellular enrichment for a compound with the K_d_ for tubulin of 0.9 μM (*S*-methyl DM1) in a cell containing 10 to100 μM tubulin. From the binding curve (Fig. 1A), and MCF7 cell volume (Table 5), we estimated that at any concentration of *S*-methyl DM1 in the medium below its apparent K_d_ value to cells, its intracellular enrichment (as a weak complex with intracellular tubulin) to be ~ 250-fold. Thus, our *in silico* simulations appear to be consistent with the experimental data for cells.

## Discussion

**Fig 6:**
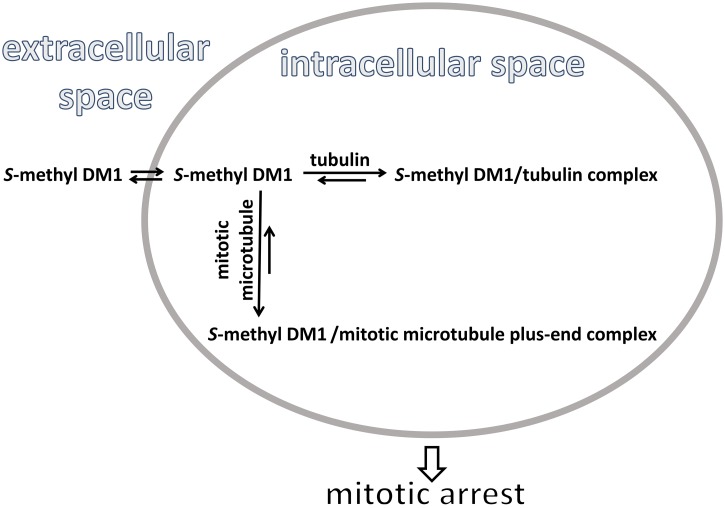
Intracellular accumulation of *S-*methyl DM1 in cells *via* its low-affinity interaction tubulin. The large number of intracellular tubulin molecules leads to accumulation of *S-*methyl DM1 inside cells at low external concentrations. *S-*methyl DM1 loosely associated with tubulin is available for binding to the tips of mitotic spindle microtubules, which leads to suppression of their dynamic instability, interfering with faithful chromosome segregation during mitosis. This in turn induces cell cycle arrest and subsequent cell death.

Our data indicate that the accumulation of maytansinoids in cells could be described as apparent affinity to intracellular binding sites. The affinity of *S*-methyl DM1 to cells (apparent K_d_ values in the range of 11 to 31 nM) and to 97 μM tubulin-loaded liposomes (apparent K_d_ of 22 nM) is higher than its affinity to either nonpolymerized tubulin in solution, or to the tips of reconstructed microtubules (apparent K_d_ of 0.4 to 0.9 μM, and 0.1 μM, respectively [6,8]). *S*-methyl DM1 does not seem to induce oligomerization of tubulin. The number of intracellular binding sites for *S*-methyl DM1 in MCF7 cells (3.2 x 10^7^), is comparable to the number of intracellular tubulin molecules (4 to 6 x 10^7^), and of intracellular binding sites for paclitaxel (5.7 x 10^7^), known to bind to assembled tubulin in ~ 1:1 ratio [23,24]. Scatchard plots indicated that *S*-methyl DM1 interacted with its intracellular binding sites uniformly, with the same affinity, suggesting that non-assembled tubulin constitutes the majority of its intracellular binding sites and is responsible for the high-affinity accumulation of *S*-methyl DM1 in cells even at very low concentrations. This notion is supported by our experiments with tubulin-containing liposomes, which showed that the low-affinity interaction of *S*-methyl DM1 with individual unassembled tubulin molecules entrapped at high concentration in liposomes was sufficient for its apparent high-affinity towards the tubulin-containing liposomes, resulting in intraliposomal accumulation. Based on our findings, we propose a model, depicted in Fig. 6, for the intracellular accumulation of maytansinoids. Maytansinoid molecules diffuse from a dilute extracellular solution into a cell, and associate with tubulin. Their interactions with individual tubulin molecules are weak, but the abundance of tubulin inside the cell favors, after dissociation of maytansinoid-tubulin complex, re-binding of the maytansinoid to a tubulin molecule over its efflux. This phenomenon of high—affinity intracellular accumulation of a compound that has only weak affinity for its intracellular target, resembles previously described “remote loading” of liposomes with organic compounds based on a transmembrane gradient of amphipathic weak acids or bases [27], and partition of a compound in a mixture of two immiscible solvents [28].

This model helps to understand the high cytotoxic potency of *S*-methyl DM1. The high intracellular concentration of weak maytansinoid-tubulin complexes generates, at any given moment, a high concentration of free maytansinoid available for binding to the tips of mitotic spindle microtubules, to which *S*-methyl DM1 has ~ 4–9-fold higher affinity than to tubulin. Association of maytansinoids with mitotic spindle microtubules leads to suppression of the microtubule dynamic instability [4,6], which in turn induces mitotic arrest. Mitotic catastrophe and subsequent cell death may occur when even a small fraction of chromosomes fail to segregate [29] further increasing the sensitivity of cells to the cytotoxic effects of maytansinoids.

Maytansinoids and vinblastine share many similarities: both bind to β-tubulin and tips of microtubules, both, at high concentrations, inhibit microtubule polymerization, both accumulate in cells, and both appear to cause cell cycle arrest and cell death *via* a similar mode—suppression of dynamic instability of microtubules [4,5,7,8,30,31]. On the other hand, while *S*-methyl DM1 and maytansine do not seem to induce significant aggregation of tubulin in cell-free systems [6,8], or oligomerization in cells (this study), vinblastine and other vinca alkaloids increase the affinity of tubulin for itself inducing its extensive oligomerization in cell-free syslems [19,32] and in cells (this study). The reasons for this difference are at present unknown.

Maytansine binding site is located on the β-tubulin subunit adjacent to the guanine-nucleotide binding site, as shown by X-ray crystallography [33]. In accord, maytansine presumably binds to a microtubule at its plus end [6], where β-subunit of tubulin is exposed [33]. Tubulin at the microtubule plus end contains GTP [4]. Cytoplasmic tubulin is a mixture of tubulin-GTP and tubulin-GDP [4, 34]. Since the affinity of maytansine to tubulin had been examined with tubulin isolated under conditions that likely result in a tubulin-GTP/tubulin-GDP mixture [6, 8, 30, 34, 35], it is not clear if the affinities of maytansine to tubulin-GTP and tubulin-GDP differ.

While some tubulin-binding agents or their conjugates with antibodies are effective as anticancer drugs [1,2,3], inhibitors of cell cycle kinases, another class of compounds that induce cell cycle arrest, produced disappointing results in the clinics [36]. The reasons for the poor clinical performance by the kinase inhibitors are at present unclear, and may relate to either their poor retention in cells, and/or the residual activity of the target kinase in their presence. One difference between these two classes of mitotic inhibitors is that while the former kill cancer cells at markedly lower concentrations than those required for their association with tubulin or microtubules [4,5], the latter are cytotoxic only at concentrations far exceeding those required for inhibiting their target kinases [37]. Our results indicate that a low-affinity interaction of a drug with an abundant intracellular protein may be sufficient for a high-affinity accumulation of the drug in cells, suggesting a novel design principle for the pharmacological enrichment of small-molecule therapeutics within cells.

## Supporting Information

S1 Fig Intracellular fluorescence autocorrelation curves for ECFP tubulin in ECFP-PTK2 cells treated with various microtubule depolymerizing agents. Each curve was the average of individual cell measurements: a total of 30 cells and 144 measurements were collected for average for non-treated cells, a total of 12 cells and 60 measurements for nocodazole, a total of 9 cells and 54 measurements for both *S*-methyl DM1 and demecolcine, and a total of 11 cells and 52 measurements for vinblastine.
(TIF)
