# Supplementary figures and images for: High-Affinity Accumulation of a Maytansinoid in Cells via Weak Tubulin Interaction
Source: PLoS One. 2015 Feb 11;10(2):e0117523. doi: 10.1371/journal.pone.0117523 (PMC4324968; doi:10.1371/journal.pone.0117523)

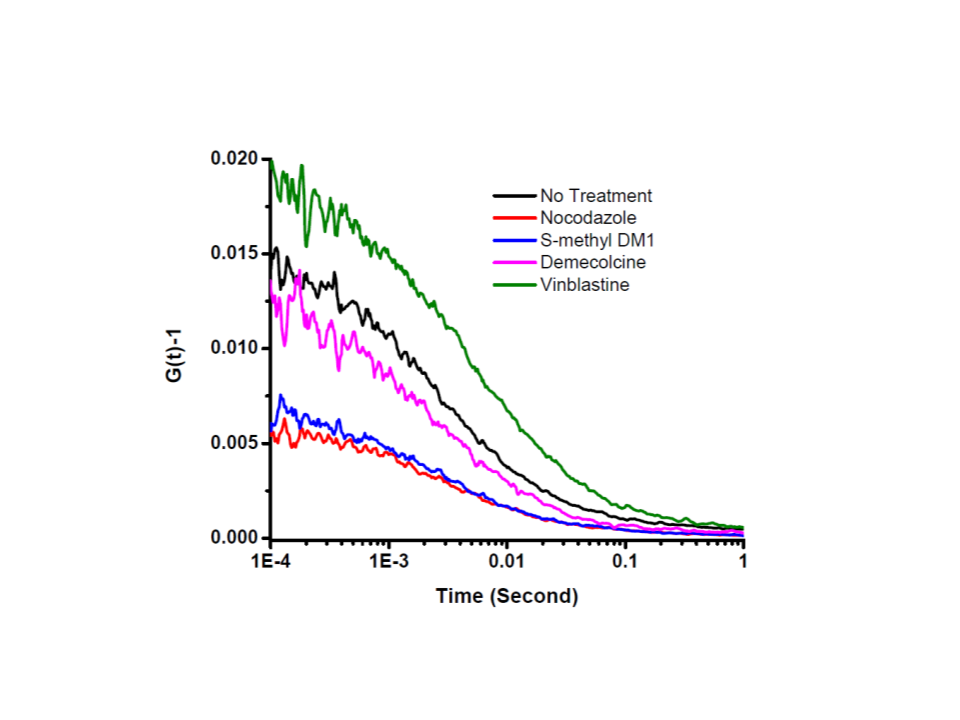

Supplement: S1 Fig — Each curve was the average of individual cell measurements: a total of 30 cells and 144 measurements were collected for average for non-treated cells, a total of 12 cells and 60 measurements for nocodazole, a total of 9 cells and 54 measurements for both S-methyl DM1 and demecolcine, and a total of 11 cells and 52 measurements for vinblastine. (TIF) [file pone.0117523.s001.tif]
